# Emergence of NDM-producing *Enterobacterales* infections in companion animals from Argentina

**DOI:** 10.1186/s12917-024-04020-z

**Published:** 2024-05-03

**Authors:** Juan Manuel de Mendieta, Andrea Argüello, María Alejandra Menocal, Melina Rapoport, Ezequiel Albornoz, Javier Más, Alejandra Corso, Diego Faccone

**Affiliations:** 1 Servicio Antimicrobianos, Laboratorio Nacional de Referencia en Resistencia a los Antimicrobianos (LNRRA), INEI-ANLIS “Dr. Carlos G. Malbrán”, Ciudad de Buenos Aires, Argentina; 2 Laboratorio Diagnotest, El Palomar, Buenos Aires, Argentina; 3 Consejo Nacional de Investigaciones Científicas y Técnicas, CONICET, Ciudad de Buenos Aires, Argentina

**Keywords:** NDM, *Enterobacterales*, Companion animals, Carbapenemase

## Abstract

Antimicrobial resistance is considered one of the most critical threat for both human and animal health. Recently, reports of infection or colonization by carbapenemase-producing *Enterobacterales* in companion animals had been described. This study report the first molecular characterization of NDM-producing *Enterobacterales* causing infections in companion animals from Argentina. Nineteen out of 3662 *Enterobacterales* isolates analyzed between October 2021 and July 2022 were resistant to carbapenemes by VITEK2C and disk diffusion method, and suspected to be carbapenemase-producers. Ten isolates were recovered from canine and nine from feline animals. Isolates were identified as *K. pneumoniae* (*n* = 9), *E. coli* (*n* = 6) and *E. cloacae* complex (*n* = 4), and all of them presented positive synergy among EDTA and carbapenems disks, mCIM/eCIM indicative of metallo-carbapenemase production and were also positive by PCR for *bla*_NDM_ gene. NDM variants were determined by Sanger sequencing method. All 19 isolates were resistant to β-lactams and aminoglycosides but remained susceptible to colistin (100%), tigecycline (95%), fosfomycin (84%), nitrofurantoin (63%), minocycline (58%), chloramphenicol (42%), doxycycline (21%), enrofloxacin (5%), ciprofloxacin (5%) and trimethoprim/sulfamethoxazole (5%). Almost all isolates (17/19) co-harbored *bla*_CTX-M_ plus *bla*_CMY_, one harbored *bla*_CTX-M_ alone and the remaining *bla*_CMY_. *E. coli* and *E. cloacae* complex isolates harbored *bla*_CTX-M-1/15_ or *bla*_CTX-M-2_ groups, while all *K. pneumoniae* harbored only *bla*_CTX-M-1/15_ genes. All *E. coli* and *E. cloacae* complex isolates harbored *bla*_NDM-1_, while in *K. pneumoniae bla*_NDM-1_ (*n* = 6), *bla*_NDM-5_ (*n* = 2), and *bla*_NDM-1_ plus *bla*_NDM-5_ (*n* = 1) were confirmed. MLST analysis revealed the following sequence types by species, *K. pneumoniae*: ST15 (*n* = 5), ST273 (*n* = 2), ST11, and ST29; *E. coli*: ST162 (*n* = 3), ST457, ST224, and ST1196; *E. cloacae* complex: ST171, ST286, ST544 and ST61. To the best of our knowledge, this is the first description of NDM-producing *E. cloacae* complex isolates recovered from cats. Even though different species and clones were observed, it is remarkable the finding of some major clones among *K. pneumoniae* and *E. coli*, as well as the circulation of NDM as the main carbapenemase. Surveillance in companion pets is needed to detect the spread of carbapenem-resistant *Enterobacterales* and to alert about the dissemination of these pathogens among pets and humans.

## Introduction

Antimicrobial resistance (AMR) is a growing and serious threat for both human and animal health. In recent years, AMR has been accelerated by the misuse and overuse of antimicrobials in human and veterinary medicine [1]. Antimicrobials are commonly indicated prophylactically and therapeutically, and as growth promoters in animal production in some countries, but only for treatment of infections in humans and companion animals [2]. Companion animals are often treated with the same or similar antibiotics that are used in human health, which poses a serious risk of selection and dissemination of pathogens and resistance mechanisms that can circulate between human and animal populations [2, 3]. This situation is aggravated by close contact between pets and their owners, which increases the risk of transmission between them [3, 4].

Particularly, carbapenem-resistant *Enterobacterales* (CRE) are considered one of the most critical problems associated with AMR [5]. Carbapenems are broad-spectrum β-lactam antibiotics used to treat serious infections caused by multidrug-resistant pathogens [6]. Infections caused by CRE have a high health burden and represent a real diagnostic and therapeutic challenge in healthcare centers [5]. While CRE have been associated with nosocomial infections in humans, there has been an increase of reports of these pathogens in veterinary medicine in recent years [7]. It is considered that the dissemination of CRE in companion animals would be through two main ways [4]: (i) by zooanthroponosis, where the direction of transmission would go from humans, colonized by these pathogens, to pets; or (ii) due to contamination of environments, mainly veterinary facilities.

Resistance to carbapenems in *Enterobacterales* is mainly mediated by carbapenemases [8]. Major groups of these enzymes include class A and D serine carbapenemases such as KPC and OXA-48-like, respectively, and class B metallo-carbapenemases such as NDM, IMP and VIM [9]. Among these, KPC, NDM and OXA-48-like carbapenemases are the most frequent worldwide in humans [9]. In Argentina, KPC and NDM are the main prevalent carbapenemases in *Enterobacterales* recovered from humans infections (http://antimicrobianos.com.ar/wp-content/uploads/2023/04/Multicenter-Prospective-Study-of-Carbapenemase-Producing-Enterobacterales-CPE-in-the-COVID-19-Era-in-Argentina-RECAPT-AR.pdf). KPC was initially described in our country in 2006 and disseminated mainly by the clonal expansion of *Klebsiella pneumoniae* ST258 [10]. The first report of NDM was in 2014 from three *Providencia rettgeri* clinical isolates, and since then it has spread to other *Enterobacterales* species [11].

NDM and OXA-48 are the most widespread carbapenemases reported in companion animals [12]. The first report of NDM date back to 2008 in the United States where NDM-1-producing *Escherichia coli* isolates were found in canine and feline samples [13]. Hereafter, other carbapenemase-producing *Enterobacterales* recovered form pets were reported worldwide [4, 10]. In Argentina, sporadic cases of carbapenem-resistant *Enterobacterales* were described in a recent retrospective surveillance study however the molecular mechanisms were not characterized [14]. To the best of our knowledge, this study represents the first molecular characterization of NDM-producing *Enterobacterales* of infections in companion animals from Argentina.

## Materials and methods

### Bacterial isolates

Between October 2021 and July 2022, 3662 *Enterobacterales* were processed at the Diagnotest Laboratory (Buenos Aires, Argentina), a veterinary clinical microbiology laboratory. Nineteen of them were resistant to carbapenemens, nine from felines and ten from canines. These isolates came from ten veterinary hospitals located in the Buenos Aires Province (*n* = 4) and Buenos Aires City (*n* = 6), and corresponding to 13 ambulatories and 6 hospitalized patients. Bacterial identification was performed by VITEK2® system (BioMérieux, Marcy-l'Étoile, France) and confirmed by MALDI-TOF (Bruker Daltonics, Bremen, Germany). Isolates were identified as *K. pneumoniae* (*n* = 9), *E. coli* (*n* = 6) and *Enterobacter cloacae* complex (*n* = 4), and recovered from urine (*n* = 11), abdominal fluid (*n* = 2), bone (*n* = 2), gallbladder (*n* = 2), abscess (*n* = 1) and lung (*n* = 1).

### Antimicrobial susceptibility

Antimicrobial susceptibility was determined by VITEK2® system using the AST-GN98 card, E-test strips (BioMérieux, Marcy-l'Étoile, France) and/or Kirby-Bauer method. Susceptibility to ampicillin, amoxicillin/clavulanic acid, cefoxitin, cefpodoxime, ceftazidime, colistin, meropenem, imipenem, ertapenem, aztreonam, amikacin, gentamicin, ciprofloxacin, doxycycline, nitrofurantoin, chloramphenicol, trimethoprim/sulfamethoxazole and minocycline were interpreted according to the Clinical and Laboratory Standard Laboratory Institute (CLSI) guidelines [15]. Resistance to colistin was screened using Colistin Agar Spot Test [16]. European Committee for Antimicrobial Susceptibility Testing (EUCAST) breakpoints were used for ceftazidime/avibactam, fosfomycin, and tigecycline [17] while veterinary breakpoints (CLSI) were used for ceftiofur, cefovecin and enrofloxacin [18]. Carbapenemase-production was screened by synergism test, placing a 10 µg-carbapenem-containing disk (meropenem and/or imipenem) 25mm apart from a 750 µg-EDTA-containing disk and a 300 µg-phenyl boronic acid-containing disk. The modified carbapenem inactivation method (mCIM) and EDTA-mCIM (eCIM) were also performed, according to the CLSI guidelines, as an additional confirmatory tests [15].

### Molecular characterization

The presence of carbapenem resistance genes (*bla*_KPC_, *bla*_NDM_, *bla*_IMP_, *bla*_VIM_, and *bla*_OXA-48-like_) was evaluated by an in-house multiplex PCR [19]. Plasmid-mediated ampC *bla*_CMY_, ESBL (*bla*_CTX-M_ and *bla*_PER-2_), and *mcr-1* genes were confirmed by an in-house triplex and a monoplex PCR, respectively [19]. 16S rRNA methyltransferases were confirmed by a multiplex PCR [20]. All PCR reactions were set up with 200 µM of dNTP’s, 1.5 mM of MgCl_2_, 1X buffer and 1U Taq polymerase (Invitrogen Massachusetts, U.S.). Amplicons were separated by electrophoresis on a 1% agarose gel stained with SYBR-Safe and recorded with the Biorad Molecular Imager Gel DocTM XR + UV system (Bio-Rad Laboratories, California, U.S.).

Genetic relatedness among the isolates was evaluated by XbaI-digested PFGE using a CHEF-DR® III System (Bio-Rad Laboratories, California, U.S.) as previously described [19]. The DNA fragments were resolved in a 1% agarose gel applying a switching time of 2.2 to 54.2 s and a voltage of 6V/cm for 20 h at 14 °C in 0.5X TBE buffer. Isolates of the same pulsetype were considered, by inference, to belong to the same sequence type (ST). *bla*_NDM_ variants were confirmed in all isolates by Sanger Sequencing (ABI PRISM 3100 o 3730, Applied Biosystems, Massachusetts, U.S.) and *bla*_CTX-M_ group by monoplex PCR. Primers NDM-in-F (5’-CTATTTACTAGGCCTCGCATT-3’) and NDM-in-R (5’-ATAAAACGCCTCTGTCACAT-3’) were used for sequencing the entire *bla*_NDM_ gene.

### Biparental conjugation

Horizontal gene transfer of *bla*_NDM_ was evaluated by biparental conjugation assay in two selected isolates from each species: M27649 and M27789 for *K. pneumoniae*, M27717 and M27987 for *E. coli* and M27828 and M27716 for *E. cloacae* complex. *E. coli* J53 (sodium azide resistant and gentamicin susceptible) was used as recipient strain. A ratio of 3:1 of donor:recipient strains were mixed on tryptic soy agar plates and incubated during 18 h at 35°C. Conjugation mix was resuspended in 1ml of physiological saline solution. Transconjugants were selected on tryptic soy agar plates supplemented with 200 µg/ml sodium azide plus 40 µg/ml gentamicin. Gentamicin was used for transconjugant selection because aminoglycoside resistance, mediated by 16S rRNA methyltransferases [21], and is generally co-linked with the *bla*_NDM_ gene. Transconjugants were identified by conventional biochemical methods and MALDI-TOF, and the *bla*_NDM_-acquisition was evaluated by the agar diffusion disks test, synergism among EDTA and carbapenem disks, and confirmed by PCR.

## Results and discussion

Among 3662 *Enterobacterales* isolates analyzed between October 2021 and July 2022, 2745 were recovered from canine and 917 from feline animals. *E. coli*, *K. pneumoniae* and *E. cloacae* represented, 58.18% (*n* = 1597), 9.36% (*n* = 257), and 4.04% (*n* = 111) of *Enterobacterales* causing infections in canines, respectively. In felines these species represented 66.74% (*n* = 612), 8.51% (*n* = 78), and 6.43% (*n* = 59), respectively. Nineteen out of 3662 *Enterobacterales*, ten from canines and nine from felines, were resistant to imipenem (MIC ≥ 8µg/ml), and were selected for further characterization. It is important to note that none of the 19 pets was previously treated with carbapenems.

The 19 isolates were resistant to all β-lactams tested, with the exception of isolate M27948, which was susceptible to aztreonam. Additionally, all isolates were resistant to gentamicin and amikacin, and remained susceptible to colistin (100%), tigecycline (95%), fosfomycin (84%), nitrofurantoin (63%), minocycline (58%), chloramphenicol (42%), doxycycline (21%), enrofloxacin (5%), ciprofloxacin (5%) and trimethoprim/sulfamethoxazole (5%) (Table 1). The synergy disks test between carbapenems and EDTA was positive and mCIM/eCIM confirmed the production of a metallo-carbapenemase. Epidemiological, phenotypic and molecular data are sumarized in Table 1.

**Table 1:** Epidemiological, phenotypic and molecular data of NDM-producing *Enterobacterales* from companion animals

**ID**	**Organism**	**Host**	**Source**	**Jurisdiction**	**Veterinary hospital**	**Isolation date**	**Hospitalized/ambulatory**	**XbaI-PFGE type**	**MLST**	**Carbapenemase gene**	** ESBL/ampC**	**Methylase gene**	**Imipenem MIC (µg/ml)**	**Antimicrobial resistance profile**^**a**^
M27738	*K. pneumoniae *	Canine	Urine	Buenos Aires City	B	1/15/22	Ambulatory	**A**	**15**	*bla*_NDM-1_	*bla*_CTX__-M 1/15_ + *bla*_CMY_	*rmt*C	≥16	CIP, ENR, GEN, AMK, SXT, CHL, DOX, MIN, NIT, FOS
M27715	*K. pneumoniae *	Feline	Urine	Buenos Aires City	G	12/9/21	Ambulatory	**A**	**15**	*bla*_NDM-1_	*bla*_CTX__-M 1/15_ + *bla*_CMY_	*rmt*C	≥16	CIP, ENR, GEN, AMK, SXT, DOX,NIT
M27649	*K. pneumoniae *	Canine	Urine	Buenos Aires	I	10/7/21	Hospitalized	**A**	**15**	*bla*_NDM-1_	*bla*_CTX__-M 1/15_ + *bla*_CMY_	*rmt*C	≥16	CIP, ENR, GEN, AMK, SXT, DOX, NIT
M28114	*K. pneumoniae *	Canine	Urine	Buenos Aires	I	6/19/22	Ambulatory	**A**	**15**	*bla*_NDM-1_	*bla*_CTX__-M 1/15_ + *bla*_CMY_	*rmt*C	≥16	CIP, ENR, GEN, AMK, SXT, DOX,NIT
M28115	*K. pneumoniae *	Canine	Abdominal fluid	Buenos Aires City	J	8/1/22	Hospitalized	**G**	**15**	*bla*_NDM-1_	*bla*_CTX__-M 1/15_ + *bla*_CMY_	*rmt*C	≥16	CIP, ENR, GEN, AMK, SXT, CHL, DOX, NIT, FOS
M27789	*K. pneumoniae *	Canine	Urine	Buenos Aires City	J	2/8/22	Ambulatory	**B**	**29**	*bla*_NDM-5_	*bla*_CTX__-M 1/15_ + *bla*_CMY_	*rmt*B	≥16	CIP, ENR, GEN, AMK, SXT
M27986	*K. pneumoniae *	Canine	Urine	Buenos Aires City	B	5/4/22	Hospitalized	**C**	**11**	*bla*_NDM-5_	*bla*_CTX__-M 1/15_	*rmt*B	≥16	CIP, ENR, GEN, AMK, SXT, CHL, DOX, MIN, NIT
M28019	*K. pneumoniae *	Canine	Abscess	Buenos Aires	A	5/20/22	Ambulatory	**D**	**273**	*bla*_NDM-1_	*bla*_CTX__-M 1/15_ + *bla*_CMY_	*rmt*C	≥16	CIP, ENR, GEN, AMK, SXT, CHL, DOX, MIN, NIT
M28018	*K. pneumoniae *	Feline	Urine	Buenos Aires	A	5/24/22	Ambulatory	**F**	**273**	*bla*_NDM-1_+ *bla*_NDM-5_	*bla*_CTX__-M 1/15_ + *bla*_CMY_	*rmt*C + *rmt*B	≥16	CIP, ENR, GEN, AMK, SXT, DOX, NIT, FOS
M27974	*E. coli*	Feline	Gallbladder	Buenos Aires City	B	4/26/22	Hospitalized	**A**	**162**	*bla*_NDM-1_	*bla*_CTX__-M 2_ + *bla*_CMY_	*rmt*C	≥16	CIP, ENR, GEN, AMK, SXT, CHL, DOX, MIN
M27987	*E. coli*	Feline	Urine	Buenos Aires	H	5/9/22	Ambulatory	**A**	**162**	*bla*_NDM-1_	*bla*_CTX__-M 2_ + *bla*_CMY_	*rmt*C	≥16	CIP, ENR, GEN, AMK, SXT, CHL, DOX, MIN
M27788	*E. coli*	Canine	Urine	Buenos Aires City	J	2/18/22	Ambulatory	**A**	**162**	*bla*_NDM-1_	*bla*_CTX__-M 2_ + *bla*_CMY_	*rmt*C	≥16	CIP, ENR, GEN, AMK, SXT, CHL, DOX, MIN
M27717	*E. coli*	Canine	Bone	Buenos Aires	F	12/4/21	Hospitalized	**B**	**457**	*bla*_NDM-1_	*bla*_CTX__-M 2_ + *bla*_CMY_	*rmt*C	≥16	GEN, AMK, SXT, DOX, MIN
M27739	*E. coli*	Feline	Urine	Buenos Aires City	B	1/6/22	Ambulatory	**C**	**224**	*bla*_NDM-1_	*bla*_CTX__-M 1/15_ + *bla*_CMY_	*rmt*C	≥16	CIP, ENR, GEN, AMK
M27948	*E. coli*	Feline	Abdominal fluid	Buenos Aires City	D	4/13/22	Hospitalized	**D**	**1196**	*bla*_NDM-1_	*bla*_CMY_	*rmt*C	≥16	CIP, ENR, GEN, AMK, SXT, CHL, DOX
M27828	*E. cloacae *complex	Canine	Gallbladder	Buenos Aires City	D	3/26/22	Ambulatory	**A**	**171**	*bla*_NDM-1_	*bla*_CTX__-M 1/15_ + *bla*_CMY_	*rmt*C	8	CIP, ENR, GEN, AMK, SXT, CHL, FOS
M27733	*E. cloacae *complex	Feline	Lung	Buenos Aires City	J	1/15/22	Ambulatory	**B**	**286**	*bla*_NDM-1_	*bla*_CTX__-M 1/15_ + *bla*_CMY_	*rmt*C	≥16	CIP, ENR, GEN, AMK, SXT, CHL, DOX, MIN, TGC, NIT
M27716	*E. cloacae *complex	Feline	Bone	Buenos Aires City	E	12/21/21	Ambulatory	**C**	**61**	*bla*_NDM-1_	*bla*_CTX__-M 1/15_ + *bla*_CMY_	*rmt*C	≥16	CIP, ENR, GEN, AMK, SXT, CHL
M27897	*E. cloacae *complex	Feline	Urine	Buenos Aires City	C	3/4/22	Ambulatory	**D**	**544**	*bla*_NDM-1_	*bla*_CTX__-M 2_ + *bla*_CMY_	*rmt*C	≥16	CIP, ENR GEN, AMK, SXT, CHL, DOX, MIN

^a^*CIP* Ciprofloxacin, *ENR* Enrofloxacin, *GEN* Gentamicin, *AMK* Amikacin, *SXT* Trimethoprim/Sulfamethoxazole, *CHL* Chloramphenicol, *DOX* Doxycycline, *MIN* Minocycline, *TGC* Tigecycline, *NIT* Nitrofurantoin, *FOS* Fosfomycin

**Fig. 1:**
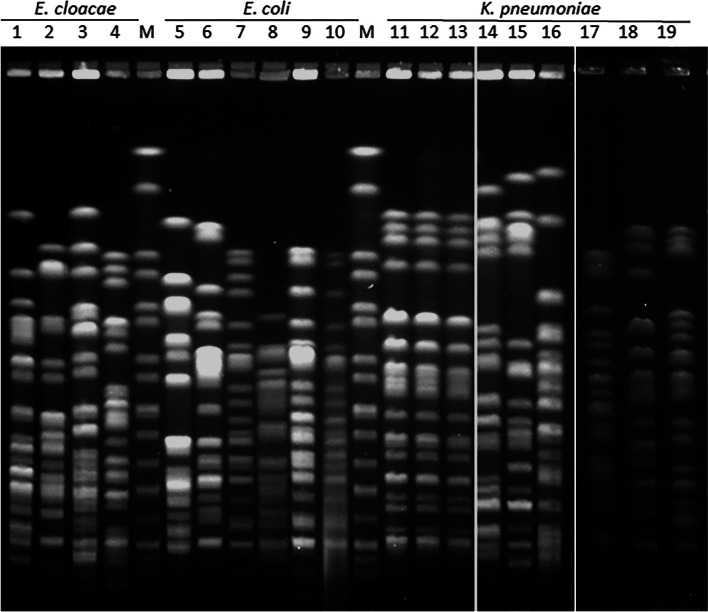
XbaI-PFGE patterns of NDM-producing Enterobacterales. 1) *E. cloacae* M27733 (B); 2) *E. cloacae* M27828 (A); 3) *E. cloacae* M27716 (C); 4) *E. cloacae* M27897 (D); M) *S*. Branderup; 5) *E. coli* M27717 (B); 6) *E. coli* M27739 (C); 7) *E. coli* M27788 (A); 8) *E. coli* M27948 (D); 9) *E. coli* M27974 (A); 10) *E. coli* M27987 (A); M) *S*. Branderup; 11) *K. pneumoniae* M27649 (A); 12) *K. pneumoniae* M27715 (A); 13) *K. pneumoniae* M27738 (A); 14 *K. pneumoniae* M27986 (C); 15) *K. pneumoniae* M28019 (D); 16) *K. pneumoniae* M28018 (F); 17) *K. pneumoniae* M27789 (B); 18) *K. pneumoniae* M28114 (A); 19) *K. pneumoniae* M28115 (G)

All isolates harbored *bla*_NDM_ and 17 out of 19 co-harbored *bla*_CTX-M_ plus *bla*_CMY_ (Table 1). One isolate harbored *bla*_CTX-M_ and the remaining *bla*_CMY_. None isolate was positive for *mcr-1* gene. PFGE analysis revealed genetic diversity among the four *E. cloacae* complex isolates (EclA, EclB, EclC and EclD), while three of the six *E. coli* isolates belonged to the same pulsetype (EcoA) and the remaining to different types (EcoB, EcoC, and EcoD) (Fig. 1; Table 1). Among the nine *K. pneumoniae* isolates, one dominant pulsetype (*n* = 4, KpnA) and five other minority ones (KpnB, KpnC, KpnD, KpnF and KpnG) were observed (Fig. 1; Table 1).

*K. pneumoniae* isolates belonged to ST15 (*n* = 5), ST273 (*n* = 2), ST11, ST29, while *E. coli* isolates belonged to ST162 (*n* = 3), ST457, ST224, and ST1196. *E. cloacae* complex isolates belonged to ST171, ST286, ST544 and ST61. All *E. coli* and *E. cloacae* complex isolates harbored *bla*_NDM-1_, while in *K. pneumoniae bla*_NDM-1_ (*n* = 6), *bla*_NDM-5_ (*n* = 2), and *bla*_NDM-1_ plus *bla*_NDM-5_ (*n* = 1) were confirmed (Table 1). *E. coli* and *E. cloacae* complex isolates harbored *bla*_CTX-M-1/15_ or *bla*_CTX-M-2_ groups, while all *K. pneumoniae* harbored only *bla*_CTX-M-1/15_ genes (Table 1). Isolates harboring *bla*_NDM-1_ were positive for rRNA methyltransferase *rmtC* gene while those with *bla*_NDM-5_, *rmtB*. Isolate M28018 harbouring *bla*_NDM-1_ and *bla*_NDM-5_ was positive for both *rmtC* and *rmtB* genes.

Conjugation assays were successfully for all six isolates evaluated, and *bla*_NDM_ harbouring plasmids were transferred to *E. coli* J53 strain. The transconjugants presented resistance to carbapenemes and aminoglycosides, positive synergy between EDTA and meropenem disks, and positive PCR for *bla*_NDM_. As has been previously reported, *bla*_NDM_ is usually associated with plasmids including in *Enterobacterales* recovered from companion animals [7, 12]. In this work, we confirmed the transfer of plasmids harboring *bla*_NDM-1_ in different clones of *E. cloacae* (EclA and EclC) and *E. coli* (EcoA and EcoB), and *bla*_NDM-1_ and *bla*_NDM-5_ in clones of *K. pneumoniae* (KpnA and KpnB), by S1-nuclease assays (data not shown).

*K. pneumoniae* was the most common species causing infections in companion animals in our collection, and contrary to *E. cloacae* and *E. coli*, this species harbors different variants of *bla*_NDM_ gene. All nine *K. pneumoniae* strains harbors *bla*_CTX-M-1/15_ group ESBL gene while eight of them were also positive for *bla*_CMY_ gene. *K. pneumoniae* M22738 belonging to clone A showed additional resistance to chloramphenicol, fosfomycin and minocycline compared with the other isolates of the same clone. It could be explained by the acquisition of extra plasmid/s coding for resistance to these drugs by this strain. ST15 CTX-M-1/15 *K. pneumoniae* lineage was previously reported in companion animals and humans in Portugal [22] and among other countries [23]. *K. pneumoniae* ST15 clone was also reported to produce different carbapenemases, however *bla*_OXA-48_ was the common [23, 24]. In our collection, 5/9 *K. pneumoniae* isolates were ST15 and were recovered from four institutions. All five isolates harbors *bla*_NDM-1_, *bla*_CTX-M-1/15_ plus *bla*_CMY_ genes, confirming the association of this lineage with CTX-M-15 and CMY. *K. pneumoniae* ST273 clone was also associated as a carbapenemase-producer, even though an isolate harbouring *bla*_NDM-1_ plus *bla*_IMP-4_ was described previously [25, 26]. Here we detected two isolates with different PFGE-patterns belonging to ST273, both harbouring *bla*_NDM-1_, but *K. pneumoniae* M28018 isolate carrying two alleles of the same gene, *bla*_NDM-1_ and *bla*_NDM-5_.

Some carbapenemases shows low hydrolytic activity against imipenem, being not a good marker to detect some carbapenemase-producer isolates [12]. Considering veterinary AST-GN98 card for VITEK2® system has only imipenem but do not include meropenem, ertapenem, or temocillin (a good phenotypic marker for screening OXA-48-like carbapenemases), there is a chance that some carbapenemases, as OXA-48-like, were overlooked. This drawback emphasizes the necessity of updating the carbapenemase screening protocols for veterinary laboratories using automated AST systems [12].

ST11 is the founder ST of CC11, a single locus variant of the hyper-epidemic ST258 clone. KPC-2-producing *K. pneumoniae* ST11 has been reported to be the dominant clone in China, South Korea and Argentina, among other countries, causing human infections [19, 27]. In companion animals, ST11 has been reported in France as a producer of plasmid-borne ampC *bla*_DHA-1_ [28] and in Germany and France as OXA-48 producer [24, 29]. *K. pneumoniae* M27986 belongs to ST11 and harbors *bla*_NDM-5_ variant and the unique isolate without the plasmid-borne ampC *bla*_CMY_ in this collection. Carbapenemase-producing *K. pneumoniae* ST29 has been reported sporadically causing human infections, but also in abattoir wastewater from Pakistan where even some strains harbors multiple carbapenemases [29]. *K. pneumoniae* M27789 belonging to ST29 was recovered from a dog´s urine sample and was positive for *bla*_NDM-5_, *bla*_CTX-M-1/15_ and *bla*_CMY_ genes. In a recent work, we described clinical *Enterobacterales* coproducing multiple carbapenemases, where *K. pneumoniae* CC307 and CC11 were the dominant clones associated with KPC-2 plus NDM-1 or KPC-2 plus NDM-5 [19]. In that report, *K. pneumoniae* ST11 (*n* = 22) and ST5995, a SLV-ST15 (*n* = 1) were detected, which evidences the circulation of these clones producing carbapenemases between humans and companion animals.

*E. coli* ST162 is a global virulent lineage that has been recovered from humans, environmental samples as well as from food, wild animals, and companion animals, and has been found associated with *bla*_CTX-M_ and/or *mcr-1* genes [30, 31]. However, a unique carbapenemase-producing *E coli* ST162 was reported and recovered from lymph nodes sample from a pygmy sperm whale (*Kogia breviceps*) [32]. Here we report three *bla*_NDM-1_-producing *E coli* ST162 recovered from cats (*n* = 2) and dog, and coproducing *bla*_CTX-M-2_ ESBL gene. All these three *E coli* ST162 isolates showed the same antimicrobial resistance profile and were recovered from different veterinary clinics.

*E. coli* ST457 has a broad host range and is a globally disseminated lineage, which has been detected in Oceania, America, Asia and Europe from humans, wild animals, companion animals and food samples, and was mainly associated with *bla*_CTX-M-1_ and *bla*_CMY-2_ [33]. In China, *E. coli* NDM-1-producing ST457 isolates associated with hemorrhagic pneumonia in mink (*Neogale* species) were found [34]. *E. coli* M27717 isolate belonging ST457 was recovered from bone sample of a dog and harbors NDM-1, CTX-M-2 and CMY β-lactamases. *E. coli* ST224 isolates has been frequently recovered in Brazil [35] and Australia [36] from animals for consumption and companion animals, harbouring *bla*_CTX-M-55_ or *bla*_CTX-M-15_ ESBLs, respectively. *E. coli* M27739 ST224 was recovered from urine from a cat and harbors NDM-1, CTX-M-1/15 and CMY β-lactamases. Finally, *E. coli* ST1196 isolates has been reported in different environments and hosts [30], producing different carbapenemases like OXA-48 in companion animals [24] and NDM-1 or KPC-2 in humans [37]. M27948 *E. coli* isolate, belonging to ST1196, recovered from a cat was the only one without a *bla*_CTX-M_ ESBL gene, but harbouring *bla*_NDM-1_ and *bla*_CMY_ genes. In a retrospective and longitudinal study of carbapenemase-producing *E. coli* isolates recovered from humans samples in Argentina [38], the major lineages observed were ST10 and ST131, however and interestingly, isolates belonging to ST457 (*n* = 1), ST224 (*n* = 1) and ST1196 (= 2) were also detected. Nevertheless, human isolates were KPC-2-producers instead of NDM-1 as was observed in pets. To our knowledge, the *E. coli* ST162 lineage, the major on this study, has not been previously described in Argentina in carbapenemase-producing *E. coli* isolates.

Carbapenemase-producing *E. cloacae* ST171 clone has been mainly reported from human samples producing KPC, NDM and OXA-48 carbapenemases, however two isolates producing KPC-4 carbapenemase and recovered from dogs were reported [39]. *E. cloacae* ST544 producing IMP-26 was reported as an epidemic clone in a tertiary hospital from China [40]. *E. cloacae* ST286 and ST61 had been found from dog samples in France (https://www.ebi.ac.uk/ena/browser/view/SAMN24584116) and environmental samples in Australia (https://www.ebi.ac.uk/ena/browser/view/SAMN10174734), respectively but not associated with carbapenemases-production. Among the four *E. cloacae* described in this work, all of them harbors *bla*_NDM-1_ variant and coproduces CTX-M and CMY β-lactamases. Only two of the *E. cloacae* clones described here were previously reported in dogs, however, and to the best of our knowledge, this is the first description of NDM-producing *E. cloacae* complex isolates recovered from cats. All isolates described here presented resistance to β-lactams including, as expected, carbapenems and ceftazidime/avibactam, aminoglycosides, ciprofloxacin and trimethoprim/sulfamethoxazole, being colistin, tigecycline and fosfomycin the most active antibiotics.

## Conclusion

We report here the emergence of NDM-producing *Enterobacterales* recovered from companion animals in Argentina. Isolates belonged to different species, being *K. pneumoniae* the most frequent NDM-producer pathogen, followed by *E. coli* and *E. cloacae* complex. Both, dissemination of carbapenem-resistant *Enterobacterales* clones and horizontal *bla*_NDM_ gene transfer mechanisms had contributed to the emergence and spread of NDM among pet isolates in our country. The finding of two NDM variants, *bla*_NDM-1_ or *bla*_NDM-5_, suggest the circulation of different plasmids, and raises the needs of characterization of these elements at molecular level. Based on all these data, we consider that it is necessary to strengthen the diagnosis, surveillance and control of carbapenem-resistant *Enterobacterales* in companion animals to prevent the dissemination of these mechanisms in the context of One Health.

## Data Availability

All data generated or analyzed during this study are included in this published article.
